# Systemic exposure following intravitreal administration of therapeutic agents: an integrated pharmacokinetic approach. 1. THR-149

**DOI:** 10.1007/s10928-021-09773-w

**Published:** 2021-07-23

**Authors:** Marc Vanhove, Bernard Noppen, Jean-Marc Wagner, Tine Van Bergen, Philippe Barbeaux, Alan W. Stitt

**Affiliations:** 1 Oxurion N.V., Gaston Geenslaan 1, 3001 Leuven, Belgium; 2 Haute École de la Province de Liège, Avenue Montesquieu 6, 4101 Seraing, Belgium; 3 Centre for Experimental Medicine, Queen’s University Belfast, Belfast, Northern Ireland, UK

**Keywords:** Intravitreal administration, Systemic exposure, Integrated pharmacokinetics

## Abstract

Intravitreal (IVT) injection of pharmacological agents is an established and widely used procedure for the treatment of many posterior segment of the eye diseases. IVT injections permit drugs to reach high concentrations in the retina whilst limiting systemic exposure. Beyond the risk of secondary complications such as intraocular infection, the potential of systemic adverse events cannot be neglected. Therefore, a detailed understanding of the rules governing systemic exposure following IVT drug administration remains a prerequisite for the evaluation and development of new pharmacological agents intended for eye delivery. We present here a novel mathematical model to describe and predict circulating drug levels following IVT in the rabbit eye, a species which is widely used for drug delivery, pharmacokinetic, and pharmacodynamic studies. The mathematical expression was derived from a pharmacokinetic model that assumes the existence of a compartment between the vitreous humor compartment itself and the systemic compartment. We show that the model accurately describes circulating levels of THR-149, a plasma kallikrein inhibitor in development for the treatment of diabetic macular edema. We hypothesize that the model based on the rabbit eye has broader relevance to the human eye and can be used to analyze systemic exposure of a variety of drugs delivered in the eye.

## Introduction

In recent years, intravitreal (IVT) injection has become the preferred administration route of pharmacological agents for the treatment of the back of the eye diseases such as age-related macular degeneration and diabetic retinopathy. Despite being invasive and associated with a low risk of rhegmatogenous retinal detachment [1], the procedure offers the advantage to immediately achieve high, therapeutically-effective drug levels in the vitreous chamber [2]. Localized IVT administration also serves to considerably limit overall systemic exposure and therefore potential systemic adverse events [2]. This, however, doesn’t imply that the risk of systemic complications doesn’t exist for drugs administered intravitreally. Indeed, the systemic risk is well illustrated by the commonly used anti-VEGF agents which IVT administration is suspected to be associated with increased risk for ischemic cerebrovascular disease, thromboembolic events, non-ocular hemorrhagic events, nephrotoxicity, acute blood pressure elevations, serious systemic infections, gastrointestinal disorders, and even mortality. This is a low but consistent risk and limiting the bioavailability of systemic VEGF could be detrimental for vascular integrity, especially in patients with retinal disease who are also at risk for cardiovascular diseases [3–7].

Therefore, accurate understanding of systemic exposure following ocular delivery remains a critical step in the development of new ophthalmological drugs. Attempts to model e.g. drug circulating levels after IVT administration remain sporadic, though, and evaluation of systemic exposure, whether for preclinical models or human data, is oftentimes restricted to analyses based solely on descriptive parameters such as maximum observed concentration (C_max_), time to reach C_max_ (t_max_), and area under the curve (AUC) [8–15]. There are, however, noticeable exceptions. Le et al. [16] described an integrated ocular-systemic model for the anti-factor D antigen-binding fragment (Fab) lampalizumab in the cynomolgus monkey which takes into account target mediated drug disposition, target turnover, and drug distribution across ocular tissues and systemic circulation. Also, Luu et al. [17] reported a model for the anti-VEGF DARPin abicipar pegol that incorporates VEGF binding kinetics, VEGF expression levels, and VEGF turnover rates to describe ocular and systemic pharmacokinetic data in the rabbit. Buitrago et al. [18] used a three-compartment pharmacokinetic model to assess plasma levels after IVT injection of the chemotherapy drug topotecan in rabbits. Gadkar et al. [19] proposed a mathematical model for ocular (including vitreous, aqueous humor, and retina) and systemic pharmacokinetic analysis of a variety of antibodies and antibody fragments that can be generalized for all protein fragments derived from an antibody regardless of the presence of an Fc region. Finally, Xu et al. [20] and Zhang et al. [21] have shown that serum concentration of the anti-VEGF Fab fragment ranibizumab after IVT administration in patients with retinal vein occlusion, diabetic macular edema, and age-related macular degeneration can be described by a one-compartment model with first-order absorption into and first-order elimination from the systemic circulation.

THR-149, a bicyclic peptide identified by a combination of phage-based selections and directed medicinal chemistry (see Teufel et al. [22] for a general description of the method), is a potent and specific inhibitor of plasma kallikrein currently being developed by Oxurion N.V. for the treatment of diabetic macular edema (source: www.clinicaltrials.gov). Here, we report the pharmacokinetic properties of THR-149 in the rabbit and propose a novel mathematical model to describe systemic exposure, in the form of circulating drug levels, following IVT administration. In the current study, the proposed mathematical expression was derived from a pharmacokinetic model that assumes the existence of an additional compartment between the vitreous humor (VH) compartment itself (i.e. the compartment where the drug is administered) and the systemic compartment. We also hypothesize, here and in our accompanying paper (Vanhove et al., this issue of J. Pharmacokinet. Pharmacodyn. 10.1007/s10928-021-09774-9), that this kind of model is generally applicable to describe plasma levels of drugs delivered into the eye.

## Methods

### Animal studies

Housing and all experimental procedures were conducted according to accepted best practice (EU Directive/AAALAC or similar) and approved by Institutional Animal Care and Research Advisory Committee of the KU Leuven according to the 2010/63/EU Directive. All animal procedures were also performed in accordance with the ARVO Statement for the Use of Animals in Ophthalmic and Vision Research.

### Nonlinear regression analyses

Nonlinear regression analyses were performed using the GraphPad Prism software ver. 5.02 (GraphPad Software Inc., La Jolla, CA) applying, unless otherwise stated, equal weighting (i.e. performing minimization based on absolute distances squared). For the modelling of plasma levels following IVT administration with Eq. 13 (Fig. 5b), and in order to compute a unique value for the parameter k_2_, the data set was analyzed “globally” i.e. considering that the data obtained for the different doses represent a unique set of data, and with k_2_ used as a “shared” parameter to employ the terminology used in GraphPad Prism (we refer the reader to the GraphPad Prism software ver. 5.02 user’s manual for a description of how global fitting of such data sets can be performed). Precision on the fitted parameters was expressed as 95% confidence intervals (CI_95%_).

### Intravitreal pharmacokinetics

Male New-Zealand White (NZW) rabbits (10 weeks old, 1.7–2.0 kg) received a single injection of 50 μL of 2.5 mg/mL THR-149 (125 µg) in each eye. General anesthesia was induced by intramuscular injection of 50 mg/mL ketamin (Ketalar, Pfizer) and 2% (v/v) sedative (Rompun, Bayer Health Care). Pupils were dilated with a drop of tropicamide (Tropicol, Théa Pharma). IVT injections were performed using 30G, 0.3 mL insulin syringes with half-unit (50 µL) marks (BD Micro-Fine) under 4 mg/mL topical anesthesia (Unicain, Thea Pharma). The animals were sacrificed 1 h, 4 h, 8 h, 24 h, 48 h, 96 h, and 168 h after injection (3 animals per time point), the injected eyes were enucleated and the vitreous was collected. The vitreous samples from each eye were treated separately, homogenized mechanically, and clarified by centrifugation as previously described [23], then further clarified by 0.2 µm filtration (Nanosep® MF with Bio-Inert® membrane, VWR, cat. 516-8554). THR-149 was quantified in the vitreous samples by HPLC. HPLC analyses were performed using an Acquity UPLC instrument (Waters). One volume of each sample was diluted into 1.5 volume of 8.33% (v/v) acetonitrile, 0.17% (v/v) trifluoroacetic acid (TFA), 0.067% (v/v) Tween-20, and 6 µL of the diluted samples were injected on a BEH300 C18 1.7 µm, 2.1 × 100 mm Acquity UPLC column (Waters, cat. 186003686) pre-equilibrated in 0.1% (v/v) TFA. Elution was performed by applying a 1-mL, 5–40% (v/v) acetonitrile linear gradient in 0.1% (v/v) TFA and THR-149 was detected by following the absorbance at 215 nm. The flow rate was 100 µL/min and the temperature of the column was maintained at 75 °C. The concentration of THR-149 in the samples was calculated by integration of the relevant peak and reference to a standard curve obtained by injection of a series of samples of known concentration.

Data (THR-149 concentration vs. time) were analyzed based on a mono-compartmental model (Eq. 1, where D is the dose, V_D,VH_ the vitreal volume of distribution, and k_1_ the first-order rate constant of vitreal drug elimination) applying a proportional weighting i.e. performing minimization based on relative distances squared. Half-life in the VH was calculated from Eq. 2 and vitreal clearance (CL_VH_) was obtained from Eq. 3.

1 $${[\mathrm{T}\mathrm{H}\mathrm{R}-149]}_{VH}=\frac{D}{{V}_{D,VH}}\cdot {e}^{-{k}_{1}\cdot t}$$

2 $${t}_{1/2}=\frac{\mathrm{l}\mathrm{n}(2)}{{k}_{1}}$$

3 $${CL}_{VH}={k}_{1}\cdot {V}_{D,VH}$$

### Intravenous pharmacokinetics

NZW rabbits (n = 8, males and females, 12 weeks old, 1.7–2.0 kg, Charles River) received a single intravenous administration of THR-149 (5 mg/kg) via the marginal ear vein using a 26G needle (Becton Dickinson) under local anesthesia (10% xylocaine spray). Blood was collected via the marginal ear vein in EDTA-coated sample tubes (Sarstedt, Multivette®, MUL-E-600) connected to a 26G needle 30 min, 2 h, and 4 h after THR-149 administration for the first four animals, and 1 h, 3 h, and 6 h after THR-149 administration for the remaining four animals. Plasma was prepared by centrifugation (10 min at 1400×*g*) and THR-149 levels were determined by LC–MS using an Acquity UPLC coupled to a QDa instrument (Waters). Prior to analysis, plasma samples were diluted with 2 or 3 volumes of 0.1% (v/v) formic acid in acetonitrile and centrifuged for 10 min at 13,000 rpm. The supernatants were further diluted in 0.1% (v/v) formic acid in water and 6 µL of the diluted samples were injected on a BEH C18 300A, 1.7 µm, 2.1 × 100 mm Acquity UPLC column (Waters, cat. 186003686) pre-equilibrated in 10% (v/v) acetonitrile and 0.1% (v/v) formic acid. The temperature of the column was maintained at 65 °C. Elution was performed by applying a 3-mL, 10–44% (v/v) acetonitrile linear gradient in 0.1% (v/v) formic acid at a flow rate of 600 µL/min. THR-149 was detected in positive polarity single ion recording mode via its [M+3H]^3+^ ion. The concentration of THR-149 in the samples was calculated by integration of the peak in the ion chromatogram and reference to a standard curve obtained by injection of a series of samples of known concentration (625–4.88 ng/mL).

Data (plasma concentration vs. time) were analyzed based on a mono-compartmental model using Eqs. 4, 5 and 6 (with D the dose, V_D,syst_ the systemic volume of distribution, k_3_ the first-order rate constant of systemic drug elimination, and CL_syst_ the systemic clearance) applying a proportional weighting.

4 $${[\mathrm{T}\mathrm{H}\mathrm{R}-149]}_{syst}=\frac{D}{{V}_{D,syst}}\cdot {e}^{-{k}_{3}\cdot t}$$

5 $${t}_{1/2}=\frac{\mathrm{l}\mathrm{n}(2)}{{k}_{3}}$$

6 $${CL}_{syst}={k}_{3}\cdot {V}_{D,syst}$$

### Plasma levels following intravitreal administration

THR-149 was solubilized at 2.5, 5.0, 7.5, 10, and 20 mg/mL. Male NZW rabbits (14–16 weeks old, average weight 3.1 kg, CEGAF) received a single, 50-µL IVT administration of THR-149 in both eyes, thus representing a dose of 0.125 mg, 0.25 mg, 0.375 mg, 0.5 mg or 1 mg per eye, depending on the concentration of the administered solution. The administration procedure was identical to the one described above except that 29G syringes were used. Blood samples were collected 0.5, 1, 4, 8, and 24 h after THR-149 administration via the marginal ear vein in EDTA-coated sample tubes (Sarstedt, Multivette®, MUL-E-600) connected to a 26G needle and plasma was prepared by centrifugation (10 min at 1400×*g*). THR-149 concentration in plasma samples was assessed by LC–MS/MS at Charles River Discovery (Groningen, The Netherlands). Of each prepared sample, a 30-µL aliquot was injected onto the HPLC column by an automated sample injector (SIL20-AC HT, Shimadzu, Japan). Chromatographic separation was performed on a reversed phase analytical column (Atlantis T3, 150 × 2.1 mm, 3.0 μm, Waters, USA) held at a temperature of 30 °C. Components were separated using a gradient of acetonitrile containing 0.1% (v/v) formic acid in ultrapurified H_2_O containing 0.1% (v/v) formic acid at a flow rate of 0.2 mL/min. The MS analyses were performed using an API 5000 MS/MS system equipped with a Turbo Ion Spray interface (both from Sciex, USA). The instrument was operated in multiple-reaction-monitoring mode. The acquisitions were performed in positive ionization mode with optimized settings for THR-149. Data were acquired and processed using the Analyst™ data system (v 1.4.2, Sciex, USA).

Data (THR-149 concentration vs. time) were analyzed based on Eqs. 7 or 13 (see ‘Results’ section).

## Results

### Intravitreal pharmacokinetics

**Fig. 1:**
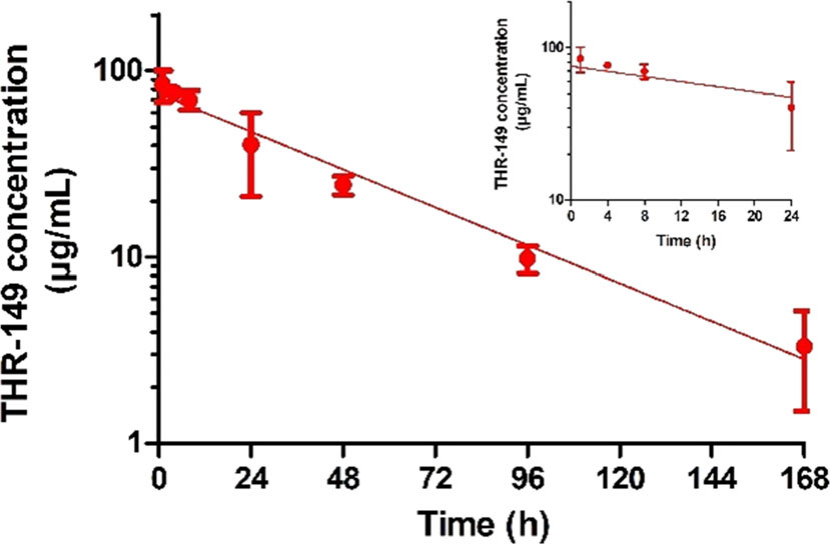
Pharmacokinetics in the rabbit VH following intravitreal administration of 125 µg of THR-149. Data are shown as mean ± SD. The solid line represents the best fit given by Eq. 1. THR-149 exhibits a relatively long residence time in the VH with a rate constant of drug elimination (k_1_) of 0.0195 h^−1^ corresponding to a half-life of 36 h

Pharmacokinetic data following administration of 125 µg of THR-149 in the rabbit eye are shown in Fig. 1 and summarized in Table 1. THR-149 exhibited a relatively long residence time in the VH with a first-order rate constant of vitreal elimination (k_1_) of 0.0195 h^−1^ corresponding to a half-life of 36 h. The vitreal volume of distribution was slightly larger than the vitreous volume (1.66 mL vs. 1.15 mL) and vitreal clearance was 0.032 mL/h. Of note, there was no evidence of metabolic degradation of THR-149 in the vitreous (not shown).

**Table 1:** Pharmacokinetic parameters of THR-149 in rabbit

Parameter	
Vitreous volume (mL)	1.15
k_1_ (h^−1^)	0.0195 [0.0177–0.0214]
Vitreal half-life (h)	36 [32–39]
V_D,VH_ (mL)	1.66 [1.46–1.92]
CL_VH_ (mL/min)	0.032 [0.029–0.036]
k_2_ (h^−1^)	0.056 [0.049–0.063]
k_3_ (h^−1^)	0.64 [0.55–0.73]
Systemic half-life (h)	1.1 [0.9–1.2]
V_D,syst_ (L/kg)	0.51 [0.40–0.73]
CL_syst_ (mL/min/kg)	5.5 [4.4–6.5]

The values of k_1_ and k_3_ were obtained from intravitreal pharmacokinetics (Fig. 1) and intravenous pharmacokinetics (Fig. 3), respectively. The value of k_2_ was calculated from plasma levels following IVT administration

### Intravenous pharmacokinetics

**Fig. 2:**
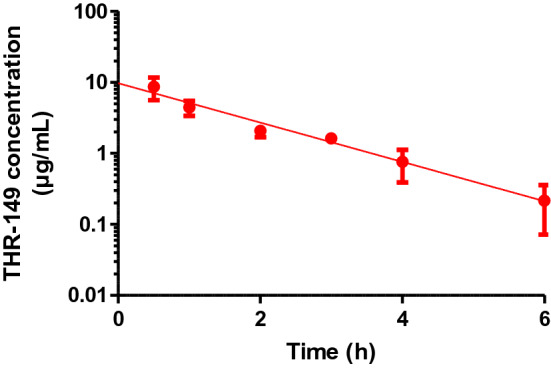
Drug plasma levels following intravenous administration of 5 mg/kg of THR-149 in rabbits. Data are shown as mean ± SD and were analyzed based on a mono-compartmental model. The solid line represents the best fit given by Eq. 4. THR-149 is cleared quickly from the circulation with a rate constant of drug elimination (k_3_) of 0.64 h^−1^ corresponding to a half-life of 1.1 h

The decrease in the circulating concentration of THR-149 following intravenous administration in rabbit (5 mg/kg) was best described by a single exponential (Fig. 2). Plasma concentration vs. time data were thus analyzed based on a mono-compartmental model (Eqs. 4, 5 and 6) leading to a first-order rate constant of systemic elimination (k_3_) of 0.64 h^−1^ (corresponding to a half-life of 1.1 h), a volume of distribution of 0.51 L/kg, and a clearance of 5.5 mL/min/kg (Table 1). Together with a high metabolic stability in presence of liver microsomes which suggests a limited potential of hepatic clearance (not shown), these data are in agreement with renal clearance at glomerular filtration rate.

### Plasma levels following intravitreal administration

**Fig. 3:**
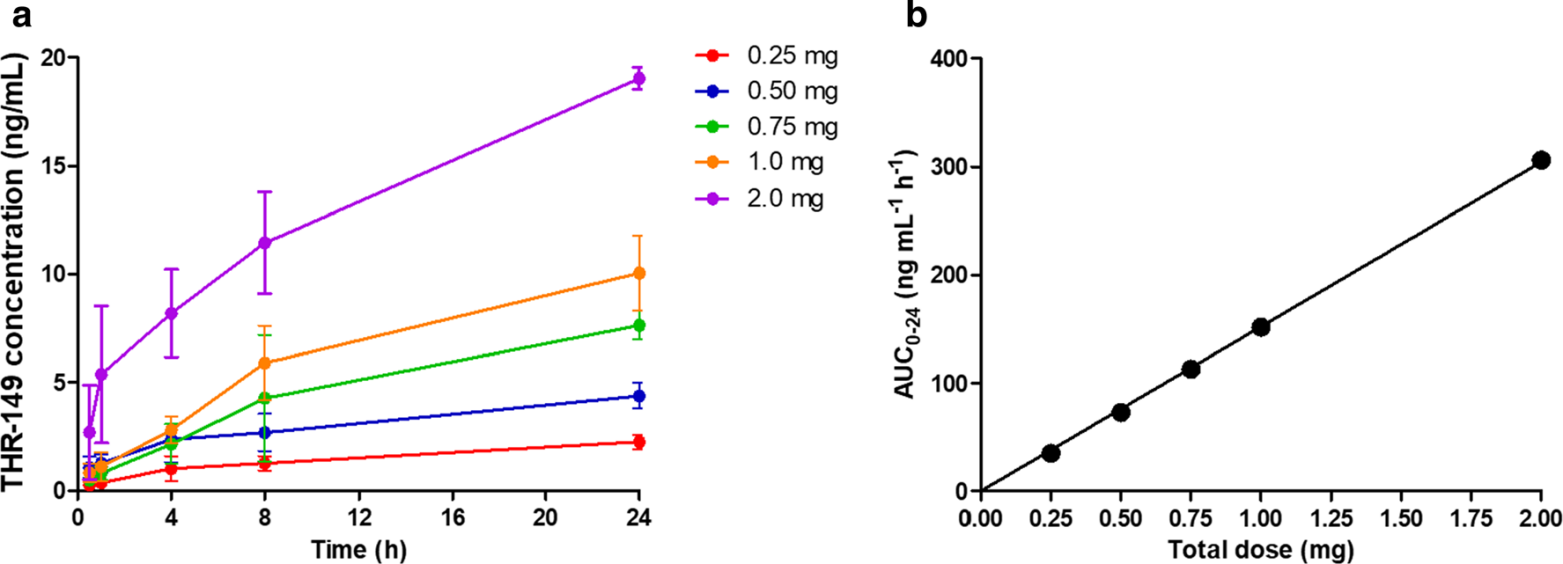
Plasma levels **(a**) and systemic exposure expressed as area under the curve (AUC) between 0 and 24 h obtained by the trapezoidal method (**b**) following bilateral intravitreal administration of THR-149 in rabbit. Data in **a** are shown as mean ± SD. Doses ranged from 0.125 to 1 mg per eye (corresponding to total doses per animal ranging from 0.25 to 2 mg)

Systemic exposure (i.e. plasma levels over time) following IVT administration of THR-149 in rabbit was measured for doses ranging from 0.125 mg per eye to 1 mg per eye (corresponding to total doses per animal ranging from 0.25 to 2 mg since THR-149 was administered bilaterally—drug elimination from each eye was assumed to be identical and additive when modeling plasma exposure). These data are presented in Fig. 3a. Plasma levels increased steadily, the higher levels being observed at the latest time point (24 h) for all doses. Overall plasma levels were also directly proportional to actual dosing as shown from the perfect correlation between the area under the curve (AUC) measured between 0 and 24 h by the trapezoidal method and the dose (Fig. 3b).

**Fig. 4:**
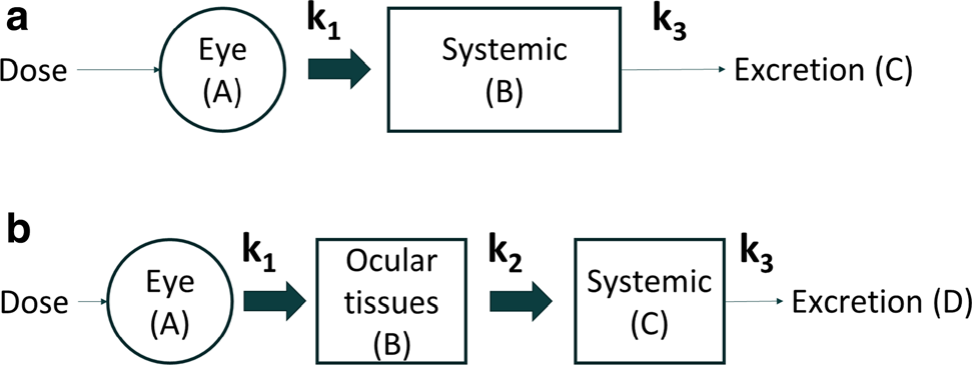
Two-compartment (**a**) and three-compartment (**b**) pharmacokinetic models used to analyze drug circulating levels following intravitreal administration of THR-149 in rabbit

Xu et al. and Zhang et al. [20, 21] have modelled circulating drug levels following IVT administration on the basis of a model with first-order absorption into and first-order elimination from the systemic circulation, a model which, therefore, is essentially identical to the two-compartment pharmacokinetic model depicted in Fig. 4a. In this model, k_1_ represents the first-order rate constant for transfer of the drug from the vitreal compartment to the systemic compartment and k_3_ the first-order rate constant for systemic elimination. Although neither Xu et al. nor Zhang et al. present an analytical solution to their model, the variation of the drug concentration in each of these compartments as a function of time can be obtained by solving (i.e. integrating) the following system of linear differential equations (Eq. syst. 1), where A and B represent the VH and systemic compartments, respectively, and C the eliminated drug (Fig. 4a):

syst. 1 $$ \begin{array}{*{20}c}    {{{dA} \mathord{\left/ {\vphantom {{dA} {dt}}} \right. \kern-\nulldelimiterspace} {dt}}} &  =  & { - k_{1}  \cdot A}  \\    {{{dB} \mathord{\left/ {\vphantom {{dB} {dt}}} \right. \kern-\nulldelimiterspace} {dt}}} &  =  & {k_{1}  \cdot A - k_{3}  \cdot B}  \\    {{{dC} \mathord{\left/ {\vphantom {{dC} {dt}}} \right. \kern-\nulldelimiterspace} {dt}}} &  =  & {k_{3}  \cdot B}  \\   \end{array}   $$

The solution of such a system of linear differential equations is common knowledge. For the second compartment, representing systemic distribution, the solution is in the form of Eq. 7 where [THR-149]_syst_ represents the concentration of the drug in plasma at any point in time (see e.g. [24]):

7 $${[\mathrm{T}\mathrm{H}\mathrm{R}-149]}_{syst}=\frac{D\cdot {k}_{1}}{{V}_{D,syst}\cdot \left({k}_{1}-{k}_{3}\right)}\cdot \left({e}^{-{k}_{3}\cdot t}-{e}^{-{k}_{1}\cdot t}\right)$$

**Fig. 5:**
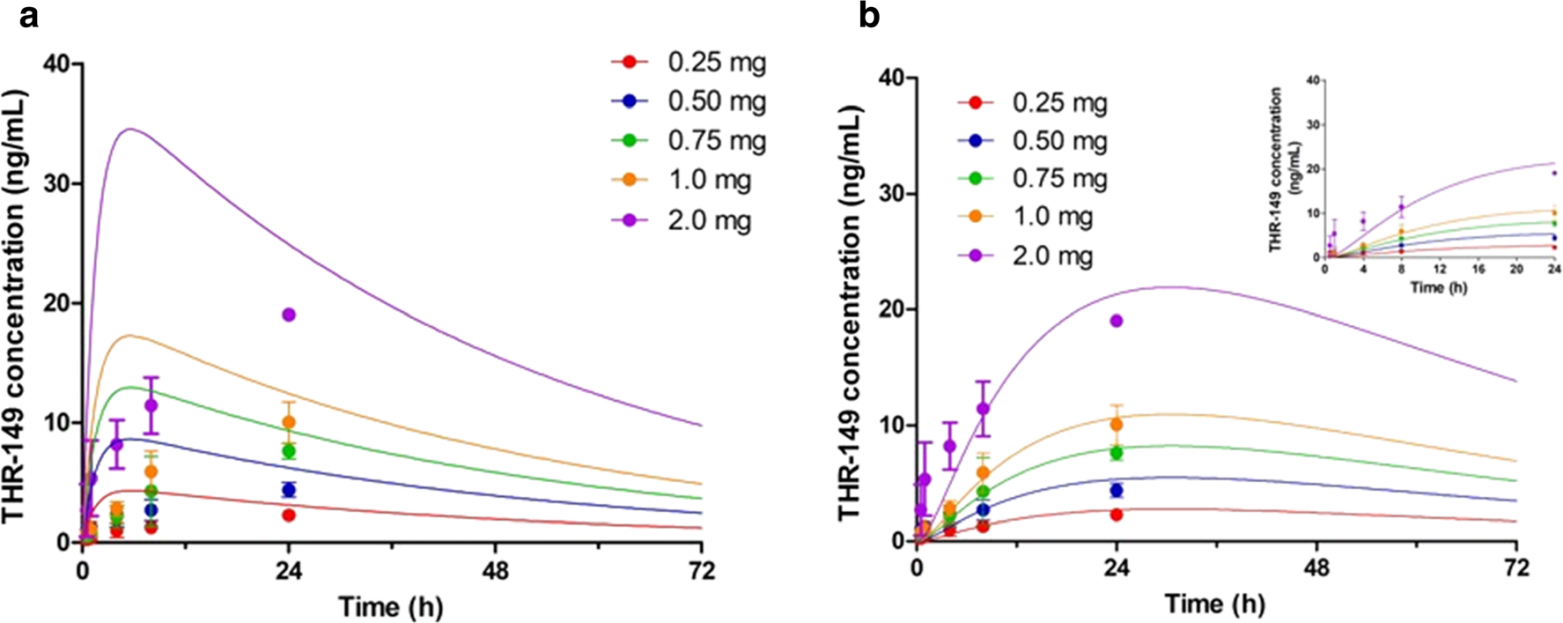
Plasma levels following bilateral intravitreal administration of THR-149 in rabbit. Data are shown as mean ± SD. Doses varied between 0.125 and 1 mg per eye (corresponding to total doses per animal ranging from 0.25 to 2 mg). Data were analyzed based either (**a**) on a two-compartment pharmacokinetic model (Fig. 4a) using Eq. 7, or (**b**) on a three-compartment pharmacokinetic model (Fig. 4b) using Eq. 13. The values of k_1_ and k_3_ in Eqs. 7 and 13 were fixed to those obtained from intravitreal and intravenous pharmacokinetics, respectively, i.e. 0.0195 h^−1^ and 0.64 h^−1^. The solid lines for (**b**) represent the best fit given by Eq. 13 with k_2_ set as the only variable parameter

The rate constants k_1_ and k_3_ in Eq. 7 also appear in Eqs. 1 and 4, respectively, and therefore the value of all the parameters of Eq. 7 (namely D, V_D,syst_, k_1_, and k_3_) are known from either intravitreal or intravenous pharmacokinetics. Equation 7 should thus accurately predict drug circulating levels following IVT administration. Figure 5a shows, however, that this is not the case. Equation 7 indeed not only poorly describes actual drug levels in general, but it also predicts that the circulating concentration will reach a maximum value (C_max_) at ~ 5.6 h whereas THR-149 levels are observed to increase steadily for at least 24 h.

We, therefore, asked whether the data could be better represented on the basis of a three-compartment pharmacokinetic model (Fig. 4b) that includes, compared to the model used above (Fig. 4a), an extra compartment that we will refer to here as the “ocular tissues compartment” and which represents a compartment through which the drug transits when being drained from the vitreous into the systemic compartment. In this model, k_2_ represents the first-order rate constant for transfer of the drug from the ocular tissues compartment to the systemic compartment.

Similarly to Eq. syst. 1, the variation of the drug concentration in each compartment as a function of time can be obtained from the following system of linear differential equations (Eq. syst. 2) where A, B, and C represent the VH, ocular tissues, and systemic compartments, respectively, and D the eliminated drug (Fig. 4b):

syst. 2 $$  \begin{array}{*{20}c}    {{{dA} \mathord{\left/ {\vphantom {{dA} {dt}}} \right. \kern-\nulldelimiterspace} {dt}}} &  =  & { - k_{1}  \cdot A}  \\    {{{dB} \mathord{\left/ {\vphantom {{dB} {dt}}} \right. \kern-\nulldelimiterspace} {dt}}} &  =  & {k_{1}  \cdot A - k_{2}  \cdot B}  \\    \begin{gathered}   {{dC} \mathord{\left/ {\vphantom {{dC} {dt}}} \right. \kern-\nulldelimiterspace} {dt}} \hfill \\   {{dD} \mathord{\left/ {\vphantom {{dD} {dt}}} \right. \kern-\nulldelimiterspace} {dt}} \hfill \\  \end{gathered}  & \begin{gathered}    =  \hfill \\    =  \hfill \\  \end{gathered}  & \begin{gathered}   k_{2}  \cdot B - k_{3}  \cdot C \hfill \\   k_{3}  \cdot C \hfill \\  \end{gathered}   \\   \end{array}  $$

Solving/integrating this system of differential equations is, however, significantly more complex and the solutions have, to our knowledge, never been reported. We propose a methodology to tackle this problem in the ‘Appendix’ section of this manuscript which leads, after final re-arrangement, to the following analytical solution (Eqs. 8, 9, 10, 11):

8 $$ A_{t}  = A_{0}  \cdot e^{{ - k_{1}  \cdot t}} $$

9 $$ B_{t}  = \frac{{A_{0}  \cdot k_{1} }}{{\left( {k_{2}  - k_{1} } \right)}} \cdot \left( {e^{{ - k_{1}  \cdot t}}  - e^{{ - k_{2}  \cdot t}} } \right) $$

10 $$ C_{t}  = \frac{{A_{0}  \cdot k_{1}  \cdot k_{2} }}{{\left( {k_{1}  - k_{2} } \right)}} \cdot \left( {\frac{1}{{k_{1}  - k_{3} }} \cdot e^{{ - k_{1}  \cdot t}}  + \frac{1}{{k_{3}  - k_{2} }} \cdot e^{{ - k_{2}  \cdot t}}  + \frac{{k_{1}  - k_{2} }}{{\left( {k_{1}  - k_{3} } \right) \cdot \left( {k_{2}  - k_{3} } \right)}} \cdot e^{{ - k_{3}  \cdot t}} } \right) $$

11 $$ D_{t}  = A_{0}  \cdot \left( \begin{gathered}   \frac{{k_{2}  \cdot k_{3} }}{{\left( {k_{2}  - k_{1} } \right) \cdot \left( {k_{1}  - k_{3} } \right)}} \cdot e^{{ - k_{1}  \cdot t}}  + \frac{{k_{1}  \cdot k_{3} }}{{\left( {k_{1}  - k_{2} } \right) \cdot \left( {k_{2}  - k_{3} } \right)}} \cdot e^{{ - k_{2}  \cdot t}}  + \frac{{k_{1}  \cdot k_{2}  \cdot \left( {k_{2}  - k_{1} } \right)}}{{\left( {k_{1}  - k_{2} } \right) \cdot \left( {k_{1}  - k_{3} } \right) \cdot \left( {k_{2}  - k_{3} } \right)}} \cdot e^{{ - k_{3}  \cdot t}}  \hfill \\   \,\,\,\,\,\,\,\,\,\,\,\,\,\,\,\,\,\,\,\,\,\,\,\,\,\,\,\,\,\,\,\,\,\,\,\,\,\,\,\,\,\,\,\,\,\,\,\,\,\,\,\,\,\,\,\, + \frac{{k_{2}  \cdot k_{3}  \cdot \left( {k_{2}  - k_{3} } \right) - k_{1}  \cdot k_{3}  \cdot \left( {k_{1}  - k_{3} } \right) + k_{1}  \cdot k_{2}  \cdot \left( {k_{1}  - k_{2} } \right)}}{{\left( {k_{1}  - k_{2} } \right) \cdot \left( {k_{1}  - k_{3} } \right) \cdot \left( {k_{2}  - k_{3} } \right)}} \hfill \\  \end{gathered}  \right) $$

In each compartment, the drug concentration depends on the dose and the volume of distribution of that compartment. Applying this reasoning to the two compartments that can be experimentally sampled, i.e. the VH and the systemic compartment (via collection of plasma), Eqs. 8 and 10 can be re-written into Eqs. 12 and 13 by substituting D/V_D_ for A_0_.

12 $$ \left[ {{\text{THR}} - 149} \right]_{{VH}}  = \frac{D}{{V_{{D,VH}} }} \cdot e^{{ - k_{1}  \cdot t}} $$

13 $$ \left[ {{\text{THR}} - 149} \right]_{{syst}}  = \frac{{D \cdot k_{1}  \cdot k_{2} }}{{V_{{D,syst}}  \cdot \left( {k_{1}  - k_{2} } \right)}} \cdot \left( {\frac{1}{{k_{1}  - k_{3} }} \cdot e^{{ - k_{1}  \cdot t}}  + \frac{1}{{k_{3}  - k_{2} }} \cdot e^{{ - k_{2}  \cdot t}}  + \frac{{k_{1}  - k_{2} }}{{\left( {k_{1}  - k_{3} } \right) \cdot \left( {k_{2}  - k_{3} } \right)}} \cdot e^{{ - k_{3}  \cdot t}} } \right) $$

**Fig. 6:**
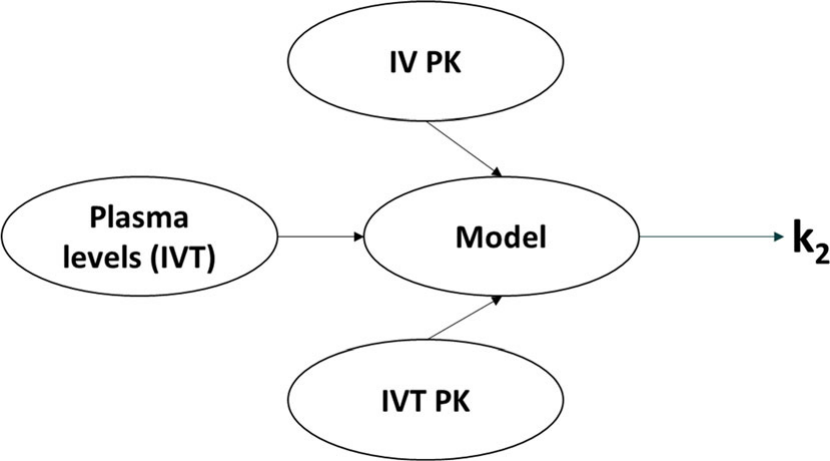
General strategy for the analysis of drug plasma levels following intravitreal administration. The mathematical model (Eq. 13) is fed with data obtained from intravenous pharmacokinetics (IV PK) and intravitreal pharmacokinetics (IVT PK) by attributing fixed values to the parameters D, V_D,syst_, k_1_, and k_3_, leaving the sole k_2_ as a variable parameter

Following a similar reasoning as above, Eq. 13 can thus be used to model or analyze plasma concentration data following IVT administration. This equation, however, is relatively complex and robust determination of all individual parameters from a given set of experimental data may be challenging. Our approach was, therefore, to “educate” this model with the information obtained from intravitreal and intravenous pharmacokinetics, as illustrated in Fig. 6, by attributing a fixed value to the parameters V_D,syst_, k_1_, and k_3_, thus leaving k_2_ as the sole variable parameter. Analysis of experimental data with this methodology are shown in Fig. 5b. It is immediately apparent that Eq. 13 allows a much better prediction of the experimental data than Eq. 7 while remaining perfectly coherent with intravitreal and intravenous pharmacokinetic data. In addition, having “educated” Eq. 13 with intravitreal and intravenous pharmacokinetic data, the value for k_2_ can be extracted with very good precision (here 0.056 h^−1^ with a 95% confidence interval of 0.049–0.063—see also Table 1) despite the complexity of the model.

**Fig. 7:**
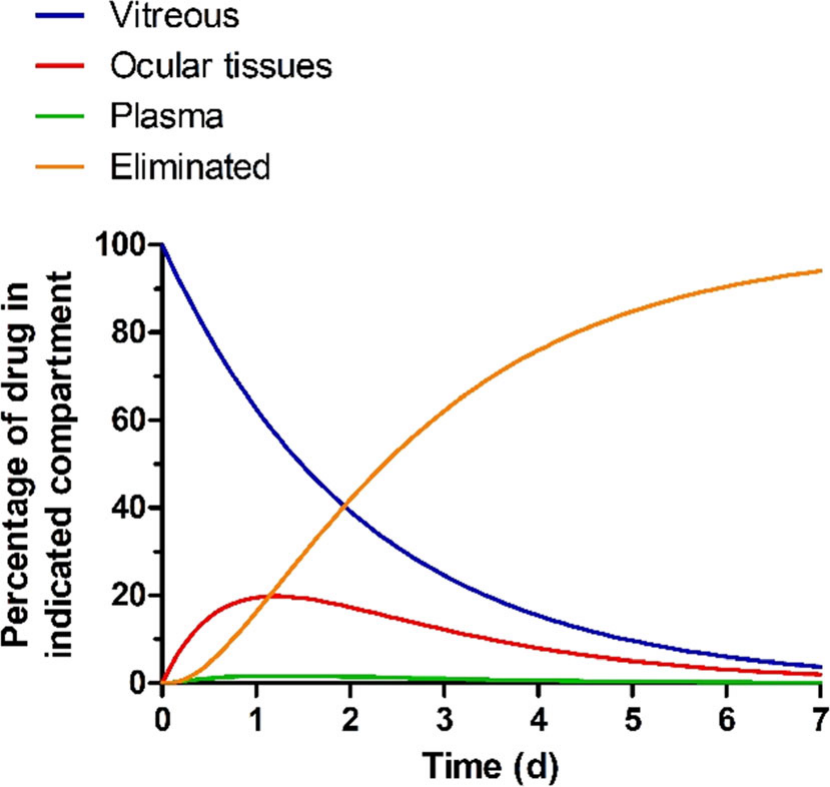
Percentage of drug present in each of the compartments (VH, ocular tissues, and systemic) and total percentage of drug eliminated following intravitreal administration of THR-149 in rabbit as predicted based on the pharmacokinetic model depicted in Fig. 4b. Calculations were based on Eqs. 8, 9, 10, 11 assuming the following values for the individual rate constants: k_1_ = 0.0195 h^−1^; k_2_ = 0.056 h^−1^; k_3_ = 0.64 h^−1^

Finally, the analytical solution of Eq. syst. 2 in the form of Eqs. 8, 9, 10 and 11 allows to calculate the fraction or percentage of drug present in each of the compartments (VH, ocular tissues, and systemic) as well as the total fraction or percentage of drug eliminated by the organism at any moment in time (Fig. 7). These simulations suggest that the fraction of drug present in the ocular tissues compartment is highest ~ 29 h post-administration, reaching ~ 20% of the total administered dose. By contrast, the percentage of drug present in the systemic compartment is predicted to be low, remaining < 2% of the total administered drug at any point in time.

### Total systemic exposure following intravenous and intravitreal administration

Total systemic exposure for intravenous or IVT administration, represented by the area under the curve (AUC) for the graph of plasma concentration vs. time, can be obtained by integrating Eq. 4 or Eq. 13 between 0 and ∞, which is straightforward for a sum of exponentials (Eq. 14).

14 $$ \int_{0}^{\infty } {\sum\nolimits_{{i = 1}}^{i} {A_{i}  \cdot e^{{ - k_{i}  \cdot t}} } }  = \sum\nolimits_{{i = 1}}^{i} {\frac{{A_{i} }}{{k_{i} }}} $$

Noteworthy, but not surprisingly, integration of Eq. 13, i.e.

15 $${\int }_{0}^{\infty }\frac{D\cdot {k}_{1}\cdot {k}_{2}}{{V}_{D,syst}\cdot \left({k}_{1}-{k}_{2}\right)}\cdot \left(\frac{1}{{k}_{1}-{k}_{3}}\cdot {e}^{-{k}_{1}\cdot t}+\frac{1}{{k}_{3}-{k}_{2}}\cdot {e}^{-{k}_{2}\cdot t}+\frac{{k}_{1}-{k}_{2}}{\left({k}_{1}-{k}_{3}\right)\cdot \left({k}_{2}-{k}_{3}\right)}\cdot {e}^{-{k}_{3}\cdot t}\right)$$

leads to

16 $${AUC}_{0-\infty }=\frac{D\cdot {k}_{1}\cdot {k}_{2}}{{V}_{D,syst}\cdot \left({k}_{1}-{k}_{2}\right)}\cdot \left(\frac{1}{{k}_{1}\cdot \left({k}_{1}-{k}_{3}\right)}+\frac{1}{{k}_{2}\cdot \left({k}_{3}-{k}_{2}\right)}+\frac{{k}_{1}-{k}_{2}}{{k}_{3}\cdot \left({k}_{1}-{k}_{3}\right)\cdot \left({k}_{2}-{k}_{3}\right)}\right)$$

an expression which is identical to the solution obtained by integrating Eq. 4:

17 $${\int }_{0}^{\infty }\frac{D}{{V}_{D,syst}}\cdot {e}^{-{k}_{3}\cdot t}=\frac{D}{{k}_{3}\cdot {V}_{D,syst}}=\frac{D}{{CL}_{syst}}$$

This demonstrates that total systemic exposure is independent of the route of administration and depends only on the dose, the systemic volume of distribution, and the rate constant of systemic elimination or, alternatively, on the dose and the systemic clearance.

### Extrapolation to human

Proper interpretation of pharmacokinetic data in preclinical models is also essential to extrapolate or predict pharmacokinetics in human. Here, the predicted clearance of THR-149 in the human VH can be determined using Eq. 18 (where the clearance is expressed in mL/h) which is based on the experimental comparison of the vitreal clearance in the rabbit and the human eye of a large number of compounds [25]. The vitreal volume of distribution of THR-149 in the human eye can be determined assuming that the ratio between the volume of distribution and the volume of vitreous is the same in the rabbit and in the human eye (Eq. 19). Based on this and on Eq. 3, one can predict a value of 0.014 h^−1^ (corresponding to a half-life of 51 h) for the rate of elimination of THR-149 in the human VH (k_1_ in model from Fig. 4b).

18 $${CL}_{human}={1.41\cdot CL}_{rabbit}+0.04$$

19 $$ \frac{{Volume\;of\;distribution\,\left( {human} \right)}}{{Vitreous\;volume\;\left( {human} \right)\,\left[ {4.36\;~{\text{mL}}} \right]}} = \frac{{Volume\;of\;distribution\,~\left( {rabbit} \right)\,\left[ {1.66\;{\text{mL}}} \right]}}{{Vitreous\;volume~\,\left( {rabbit} \right)\,\left[ {1.15\;{\text{mL}}} \right]}} $$

The rate of systemic elimination of THR-149 in human (k_3_ in model from Fig. 4b) can be estimated assuming renal clearance at glomerular filtration rate (120 mL/min for a 70 kg individual) and a systemic volume of distribution identical to the one measured in the rabbit, i.e. 0.51 L/kg. Using Eq. 6 one can then calculate a value of k_3_ of 0.20 h^−1^.

**Fig. 8:**
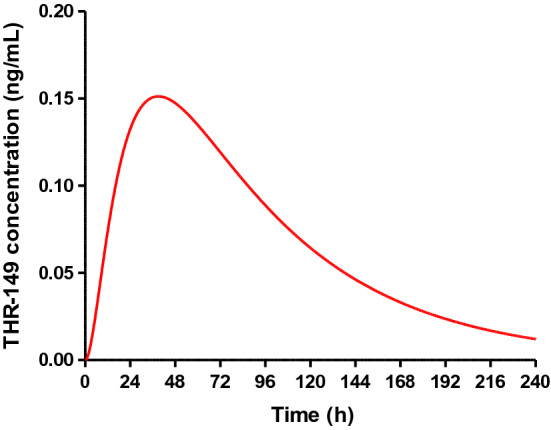
Plasma levels following intravitreal administration of 125 µg of THR-149 in human as predicted by the pharmacokinetic model shown in Fig. 4b and represented by Eq. 13. Parameters used for the calculations were as follows: body weight = 70 kg; volume of distribution = 36 L (0.51 L/kg); k_1_ = 0.014 h^−1^; k_2_ = 0.056 h^−1^; k_3_ = 0.20 h^−1^

Based on the above numbers, it is thus possible to predict THR-149 plasma levels in human following IVT administration using Eq. 13. The only unknown is the value of the first-order rate constant for the elimination of the drug from the ocular tissues compartment (k_2_ in model from Fig. 4b) in humans. Depending on the availability of specific information, it might be relevant to extrapolate the value of k_2_ in human based e.g. on the difference in trabecular mesh outflow or retinal blood flow between the two species. In the present case, though, there is no evidence that k_2_ represents aqueous humor (AH)-to-plasma transfer since AH-to-plasma transfer rate constants have been reported either for small molecules or biologics that are significantly larger than our value of k_2_ [17, 18]. Extrapolation of k_2_ to human based on trabecular mesh outflow thus doesn’t seem appropriate. Similarly, since THR-149 elimination via the posterior route appears limited (see discussion section), extrapolation of k_2_ to human based on retinal blood flow seems equally inappropriate. In the absence of a solid rationale to extrapolate that rate constant from the data collected in rabbit, we thus chose to attribute the same value of k_2_ in the two species. Predicted plasma levels following monolateral IVT administration of 125 µg of THR-149 (the highest dose tested in the clinic) are shown in Fig. 8. Plasma concentration is expected to reach a maximum value of only 0.15 ng/mL 39 h post-administration before decaying slowly. Predicted total systemic exposure is calculated to be 17.5 (ng/mL) h based on Eq. 16.

## Discussion

Bicyclic peptides are constrained peptides consisting of a peptide sequence containing 3 cysteine residues which are covalently linked to a thiol-reactive molecular scaffold [26, 27]. With a molecular weight of ~ 1.7 kDa, THR-149 thus lies between conventional small molecule drugs and macromolecules/biologics. At 0.032 mL/h, the vitreal clearance of THR-149 in the rabbit eye is relatively low, being at the very low end of the vitreal clearance spectrum observed for small molecules and at the higher end of the vitreal clearance spectrum observed for macromolecules, which may indicate dominant drug elimination via the anterior route and limited drug elimination via the posterior route (blood-retinal barrier) [25, 28]. The vitreal volume of distribution following IVT administration in rabbit was found to be well within the narrow range of 0.72–3.6 mL reported for a variety of molecules [25]. By contrast, with a half-life of only 1.1 h, systemic elimination of THR-149 in the rabbit is fast. The volume of distribution (0.51 L/kg) suggests distribution in total body water. Systemic clearance of THR-149 is 5.5 mL/min/kg which aligns with systemic pharmacokinetics in the rat (not shown) and is consistent with renal clearance [29].

Several approaches have been used to describe drug circulating levels following IVT administration. Some of the proposed models, however, are highly specific and/or elaborated and their complexity requires the use of specific software such as NONMEM (ICON Development Solutions, Dublin, Ireland) or SimBiology (MathWorks, Inc., Natick, MA, USA). Therefore, by its simplicity, a two-compartment model such as proposed by Xu et al. and Zhang et al. [20, 21] (Fig. 4a) may arguably appear as the most generally applicable. This model, however, proved inadequate to describe THR-149 circulating levels following IVT administration in the rabbit. By contrast, experimental data could be accurately represented on the basis of a three-compartment model that assumes the existence of an additional compartment localized between the VH compartment and the systemic compartment (Fig. 4b). Here, we have referred to the additional compartment as the “ocular tissues” compartment. Worth noting, however, and because of the very nature of compartmental pharmacokinetic modelling, we cannot speculate as to where the drug exactly physically distributes in that compartment; hence the model only provides information on the drug distribution between the different compartments over time as well as on the rates of drug transfer from one compartment to the next.

Intuitively, because the vitreous is not in direct physical contact with the plasma, the need to consider the existence of an ocular tissues compartment to accurately describe the evolution of plasma levels following IVT administration is not incongruous. In addition, we report in our accompanying paper a similar observation in the rabbit and the pig for THR-687, a pan-integrin antagonist currently in development for the treatment of diabetic macular edema. We, therefore, hypothesize that the reasoning used here applies to a majority of drugs administered intravitreally and that circulating levels could be analyzed with the model proposed here, at least for drugs which systemic elimination obeys mono-compartmental pharmacokinetics (for drugs which systemic elimination obeys bi-compartmental pharmacokinetics, we refer the reader to our accompanying paper).

Another significant advantage of the methodology depicted here lies in the fact that we provide an analytical solution to the system of linear differential equations that describes the proposed model, which eliminates the need for complex software’s such as those mentioned above and allows data analysis by standard nonlinear regression analysis. Of note, it is conceivable that data analysis using Eq. 13 would return a value of k_2_ much larger than the one of k_1_. This, however, would not invalidate the general concept proposed here but rather simply indicate that the tested drug doesn’t accumulate significantly in the ocular tissues compartment. To be observed, Eq. 13 simplifies into Eq. 7 when k_2_ ≫ k_1_ & k_3_.

Although accurately describing THR-149 circulating levels following intravitreal administration, it is, however, necessary to mention that one aspect that the proposed model does not incorporate is binding to the drug target. Drug-target interaction can potentially modify the overall drug elimination and other researchers have very elegantly addressed the matter (see e.g. [16, 17]). However, the impact of target binding on overall drug elimination can only become significant when the drug levels and the target concentration are of the same order of magnitude. Given that intravitreal administration results in high drug levels in the vitreous chamber [2], it is likely that in a majority of cases the drug concentration will be much larger than the target concentration, making it possible to neglect drug-target interaction in pharmacokinetic modelling.

Also, rather than the empirical approach used here which assumes the existence of a unique and undefined ocular tissues compartment, other pharmacokinetic models have been proposed that incorporate more physiological/morphological aspects of drug ocular elimination and distribution. E.g. Gadkar et al., (2015), Le et al., (2015), and Luu et al., (2020) have reported sophisticated models that account for drug distribution within the retina (or retina and choroid) and diffusion to the aqueous humor [16, 17, 19], and Buitrago et al. have applied a model which allows the drug to reach the circulation both from the vitreous and from the aqueous humor, thereby accounting for anterior and posterior ocular clearance [18]. However, given the differences in structure and assumptions made between our proposition and these different models, additional studies applied to various types of drugs will be necessary to compare these models for their ability to accurately predict drug circulating levels following ocular administration.

## Conclusion

We describe, here and in our accompanying paper, pharmacokinetic models for the analysis and prediction of plasma levels following IVT administration and hypothesize that these models will apply to a variety of drugs administered in the eye.

## Appendix

We propose the following methodology to solve/integrate Eq. syst. A.

syst. A $$ \begin{array}{*{20}c}    {{{dA} \mathord{\left/ {\vphantom {{dA} {dt}}} \right. \kern-\nulldelimiterspace} {dt}}} &  =  & { - k_{1}  \cdot A}  \\    {{{dB} \mathord{\left/ {\vphantom {{dB} {dt}}} \right. \kern-\nulldelimiterspace} {dt}}} &  =  & {k_{1}  \cdot A - k_{2}  \cdot B}  \\    \begin{gathered}   {{dC} \mathord{\left/ {\vphantom {{dC} {dt}}} \right. \kern-\nulldelimiterspace} {dt}} \hfill \\   {{dD} \mathord{\left/ {\vphantom {{dD} {dt}}} \right. \kern-\nulldelimiterspace} {dt}} \hfill \\  \end{gathered}  & \begin{gathered}    =  \hfill \\    =  \hfill \\  \end{gathered}  & \begin{gathered}   k_{2}  \cdot B - k_{3}  \cdot C \hfill \\   k_{3}  \cdot C \hfill \\  \end{gathered}   \\   \end{array}  $$

The first step is to re-arrange the system of differential equations in the form of Eq. A:

A $$ \left( {\begin{array}{*{20}c}    {{{dA} \mathord{\left/ {\vphantom {{dA} {dt}}} \right. \kern-\nulldelimiterspace} {dt}}}  \\    {{{dB} \mathord{\left/ {\vphantom {{dB} {dt}}} \right. \kern-\nulldelimiterspace} {dt}}}  \\    {{{dC} \mathord{\left/ {\vphantom {{dC} {dt}}} \right. \kern-\nulldelimiterspace} {dt}}}  \\    {{{dD} \mathord{\left/ {\vphantom {{dD} {dt}}} \right. \kern-\nulldelimiterspace} {dt}}}  \\   \end{array} } \right) = \left( {\begin{array}{*{20}c}    { - k_{1} }  \\    {k_{1} }  \\    0  \\    0  \\   \end{array} ~~~~~\begin{array}{*{20}c}    0  \\    { - k_{2} }  \\    {k_{2} }  \\    0  \\   \end{array} ~~~~\begin{array}{*{20}c}    0  \\    0  \\    { - k_{3} }  \\    {k_{3} }  \\   \end{array} ~~~~~~\begin{array}{*{20}c}    0  \\    0  \\    0  \\    0  \\   \end{array} } \right)\left( {\begin{array}{*{20}c}    A  \\    B  \\    C  \\    D  \\   \end{array} } \right) $$

Let’s call it the primary system and let’s call X the column vector of the unknowns.

B $$X=\left(\begin{array}{c}A\\ B\\ C\\ D\end{array}\right)$$

and

C $$\dot{X}=\left(\begin{array}{c}dA/dt\\ dB/dt\\ dC/dt\\ dD/dt\end{array}\right)$$

its temporal derivative. Let’s also call

D $$ S = \left( {\begin{array}{*{20}c}    { - k_{1} }  \\    {k_{1} }  \\    0  \\    0  \\   \end{array} ~~~~~\begin{array}{*{20}c}    0  \\    { - k_{2} }  \\    {k_{2} }  \\    0  \\   \end{array} ~~~~\begin{array}{*{20}c}    0  \\    0  \\    { - k_{3} }  \\    {k_{3} }  \\   \end{array} ~~~~~~\begin{array}{*{20}c}    0  \\    0  \\    0  \\    0  \\   \end{array} } \right) $$

the primary system matrix. The primary system of differential equations can then be written in matrix form:

E $$ \dot{X} = S \cdot X $$

The four differential equations of the primary system (Eq. syst. A) are coupled. This means that the derivative of one unknown depends on one or several other unknowns. For example, dB/dt depends on B with the coefficient − k_2_ but also on A with the coefficient k_1_. The reasoning here is to de-couple the four equations so that the derivative of each unknown only depends on itself. The mathematical technique we used is the diagonalization of the matrix S so that we can write:

F $$ S = P~D~P^{{ - 1}} $$

where D is a diagonal matrix containing the eigenvalues of the matrix S, i.e.:

G $$ D = \left( {\begin{array}{*{20}c}    {\lambda _{1} }  \\    0  \\    0  \\    0  \\   \end{array} ~~~~~\begin{array}{*{20}c}    0  \\    {\lambda _{2} }  \\    0  \\    0  \\   \end{array} ~~~~\begin{array}{*{20}c}    0  \\    0  \\    {\lambda _{3} }  \\    0  \\   \end{array} ~~~~~~\begin{array}{*{20}c}    0  \\    0  \\    0  \\    {\lambda _{4} }  \\   \end{array} } \right) $$

and P is a squared matrix where the columns are the eigenvectors of S corresponding to the eigenvalues λ_1_, λ_2_, λ_3_, and λ_4_, in the same order. P^−1^ is the inverse matrix of P. The eigenvalues of the matrix S are the four solutions (roots) of the characteristic equation of S:

H $$ dtm\left( {S - ~\lambda I} \right) = 0 $$

where dtm is the determinant of the matrix and I the identity matrix. Here, λ_1_ = − k_1_, λ_2_ = − k_2_, λ_3_ = − k_3_, and λ_4_ = 0 so that

I $$ D = \left( {\begin{array}{*{20}c}    { - {\text{k}}_{1} }  \\    0  \\    0  \\    0  \\   \end{array} ~~~~~\begin{array}{*{20}c}    0  \\    { - {\text{k}}_{2} }  \\    0  \\    0  \\   \end{array} ~~~~\begin{array}{*{20}c}    0  \\    0  \\    { - {\text{k}}_{3} }  \\    0  \\   \end{array} ~~~~~~\begin{array}{*{20}c}    0  \\    0  \\    0  \\    0  \\   \end{array} } \right) $$

The eigenvectors v_i_ associated to each eigenvalue λ_i_ (i = 1, 2, 3, 4) are the solutions of the system

J $$ S~v_{i}  = \lambda _{i} ~v_{i} ~~~\left( {i = 1,2,3,4} \right) $$

In our context, this leads to:

K $$ v_{1}  = \left( {\begin{array}{*{20}c}    {{{ - \left( {k_{1}  - k_{2} } \right)\left( {k_{1}  - k_{3} } \right)} \mathord{\left/ {\vphantom {{ - \left( {k_{1}  - k_{2} } \right)\left( {k_{1}  - k_{3} } \right)} {\left( {k_{2} k_{3} } \right)}}} \right. \kern-\nulldelimiterspace} {\left( {k_{2} k_{3} } \right)}}}  \\    {{{k_{1} \left( {k_{1}  - k_{3} } \right)} \mathord{\left/ {\vphantom {{k_{1} \left( {k_{1}  - k_{3} } \right)} {\left( {k_{2} k_{3} } \right)}}} \right. \kern-\nulldelimiterspace} {\left( {k_{2} k_{3} } \right)}}}  \\    {{{ - k_{1} } \mathord{\left/ {\vphantom {{ - k_{1} } {k_{3} }}} \right. \kern-\nulldelimiterspace} {k_{3} }}}  \\    1  \\   \end{array} } \right),v_{2}  = \left( {\begin{array}{*{20}c}    0  \\    {{{\left( {k_{2}  - k_{3} } \right)} \mathord{\left/ {\vphantom {{\left( {k_{2}  - k_{3} } \right)} {k_{3} }}} \right. \kern-\nulldelimiterspace} {k_{3} }}}  \\    {{{ - k_{2} } \mathord{\left/ {\vphantom {{ - k_{2} } {k_{3} }}} \right. \kern-\nulldelimiterspace} {k_{3} }}}  \\    1  \\   \end{array} } \right),v_{3}  = \left( {\begin{array}{*{20}c}    0  \\    0  \\    { - 1}  \\    1  \\   \end{array} } \right),~v_{4}  = \left( {\begin{array}{*{20}c}    0  \\    0  \\    0  \\    1  \\   \end{array} } \right) $$

so that

L $$ P = \left( {\begin{array}{*{20}c}    {{{ - \left( {k_{1}  - k_{2} } \right)\left( {k_{1}  - k_{3} } \right)} \mathord{\left/ {\vphantom {{ - \left( {k_{1}  - k_{2} } \right)\left( {k_{1}  - k_{3} } \right)} {\left( {k_{2} k_{3} } \right)}}} \right. \kern-\nulldelimiterspace} {\left( {k_{2} k_{3} } \right)}}} & 0 & 0 & 0  \\    {{{k_{1} \left( {k_{1}  - k_{3} } \right)} \mathord{\left/ {\vphantom {{k_{1} \left( {k_{1}  - k_{3} } \right)} {\left( {k_{2} k_{3} } \right)}}} \right. \kern-\nulldelimiterspace} {\left( {k_{2} k_{3} } \right)}}} & {{{\left( {k_{2}  - k_{3} } \right)} \mathord{\left/ {\vphantom {{\left( {k_{2}  - k_{3} } \right)} {k_{3} }}} \right. \kern-\nulldelimiterspace} {k_{3} }}} & 0 & 0  \\    {{{ - k_{1} } \mathord{\left/ {\vphantom {{ - k_{1} } {k_{3} }}} \right. \kern-\nulldelimiterspace} {k_{3} }}} & {{{ - k_{2} } \mathord{\left/ {\vphantom {{ - k_{2} } {k_{3} }}} \right. \kern-\nulldelimiterspace} {k_{3} }}} & { - 1} & 0  \\    1 & 1 & 1 & 1  \\   \end{array} } \right) $$

To be noted, P only exists if k_2_.k_3_ ≠ 0 and P^−1^ only exists if k_1_ ≠ k_2_, k_1_ ≠ k_3_, and k_2_ ≠ k_3_. If k_1_ = k_2_ and/or k_1_ = k_3_ and/or k_2_ = k_3_, the matrix S is not diagonalizable and the methodology described here is not applicable.

Including Eq. F into Eq. E leads to

M $$\dot{X}=PD{P}^{-1}X$$

Multiplying the two sides of this last equality by P^−1^ leads to:

N $${P}^{-1}\dot{X}={P}^{-1}PD{P}^{-1}X\leftrightarrow \dot{\left({P}^{-1}X\right)}=D\left({P}^{-1}X\right)\leftrightarrow \dot{Y}=DY$$

where $\dot{\left({P}^{-1}X\right)}$ is equal to $\left({P}^{-1}\dot{X}\right)$ since P^−1^ is a constant matrix and where we introduced

O $$Y={P}^{-1}X=\left(\begin{array}{c}A\text{'}\\ B\text{'}\\ C\text{'}\\ D\text{'}\end{array}\right)$$

Here, A′, B′, C′, and D′ are the four secondary unknowns from which one can deduce the four primary unknowns A, B, C, and D using

P $$\left(\begin{array}{c}A\\ B\\ C\\ D\end{array}\right)=X=PY=P\left(\begin{array}{c}A\text{'}\\ B\text{'}\\ C\text{'}\\ D\text{'}\end{array}\right)$$

The secondary system of differential equations

Q $$\dot{Y}=DY$$

which can also be written

R $$ \left( {\begin{array}{*{20}c}    {{{dA^{\prime}} \mathord{\left/ {\vphantom {{dA^{\prime}} {dt}}} \right. \kern-\nulldelimiterspace} {dt}}}  \\    {{{dB^{\prime}} \mathord{\left/ {\vphantom {{dB^{\prime}} {dt}}} \right. \kern-\nulldelimiterspace} {dt}}}  \\    {{{dC^{\prime}} \mathord{\left/ {\vphantom {{dC^{\prime}} {dt}}} \right. \kern-\nulldelimiterspace} {dt}}}  \\    {{{dD^{\prime}} \mathord{\left/ {\vphantom {{dD^{\prime}} {dt}}} \right. \kern-\nulldelimiterspace} {dt}}}  \\   \end{array} } \right) = \left( {\begin{array}{*{20}c}    {\lambda _{1} }  \\    0  \\    0  \\    0  \\   \end{array} ~~~~~\begin{array}{*{20}c}    0  \\    {\lambda _{2} }  \\    0  \\    0  \\   \end{array} ~~~~\begin{array}{*{20}c}    0  \\    0  \\    {\lambda _{3} }  \\    0  \\   \end{array} ~~~~~~\begin{array}{*{20}c}    0  \\    0  \\    0  \\    {\lambda _{4} }  \\   \end{array} } \right)\left( {\begin{array}{*{20}c}    {A^{\prime}}  \\    {B^{\prime}}  \\    {C^{\prime}}  \\    {D^{\prime}}  \\   \end{array} } \right) $$

is now de-coupled and each of the four secondary differential equations can be solved/integrated separately with:

syst. B $$ \begin{array}{*{20}c}    {A^{\prime} = C_{1}  \cdot e^{{\lambda _{1}  \cdot t}}  = C_{1}  \cdot e^{{ - k_{1}  \cdot t}} }  \\    {B^{\prime} = C_{2}  \cdot e^{{\lambda _{2}  \cdot t}}  = C_{2}  \cdot e^{{ - k_{2}  \cdot t}} }  \\    {C^{\prime} = C_{3}  \cdot e^{{\lambda _{3}  \cdot t}}  = C_{3}  \cdot e^{{ - k_{3}  \cdot t}} }  \\    {D^{\prime} = C_{4}  \cdot e^{{\lambda _{4}  \cdot t}}  = C_{4} }  \\   \end{array} $$

where C_1_, C_2_, C_3_, and C_4_ are constants that will be fixed based on the initial conditions of the primary system. Introducing A′, B′, C′, and D′ in Eq. P and using Eq. L of matrix P leads to:

S $$ \left( {\begin{array}{*{20}c}    A  \\    B  \\    C  \\    D  \\   \end{array} } \right) = \left( {\begin{array}{*{20}c}    {{{ - \left( {k_{1}  - k_{2} } \right)\left( {k_{1}  - k_{3} } \right)} \mathord{\left/ {\vphantom {{ - \left( {k_{1}  - k_{2} } \right)\left( {k_{1}  - k_{3} } \right)} {\left( {k_{2} k_{3} } \right)}}} \right. \kern-\nulldelimiterspace} {\left( {k_{2} k_{3} } \right)}}} & 0 & 0 & 0  \\    {{{k_{1} \left( {k_{1}  - k_{3} } \right)} \mathord{\left/ {\vphantom {{k_{1} \left( {k_{1}  - k_{3} } \right)} {\left( {k_{2} k_{3} } \right)}}} \right. \kern-\nulldelimiterspace} {\left( {k_{2} k_{3} } \right)}}} & {{{\left( {k_{2}  - k_{3} } \right)} \mathord{\left/ {\vphantom {{\left( {k_{2}  - k_{3} } \right)} {k_{3} }}} \right. \kern-\nulldelimiterspace} {k_{3} }}} & 0 & 0  \\    {{{ - k_{1} } \mathord{\left/ {\vphantom {{ - k_{1} } {k_{3} }}} \right. \kern-\nulldelimiterspace} {k_{3} }}} & {{{ - k_{2} } \mathord{\left/ {\vphantom {{ - k_{2} } {k_{3} }}} \right. \kern-\nulldelimiterspace} {k_{3} }}} & { - 1} & 0  \\    1 & 1 & 1 & 1  \\   \end{array} } \right)\left( {\begin{array}{*{20}c}    {C_{1}  \cdot e^{{ - k_{1}  \cdot t}} }  \\    {C_{2}  \cdot e^{{ - k_{2}  \cdot t}} }  \\    {C_{3}  \cdot e^{{ - k_{3}  \cdot t}} }  \\    {C_{4} }  \\   \end{array} } \right) $$

or

syst. C $$ \begin{array}{*{20}c}    {A_{t} } &  =  & { - C_{1}  \cdot \frac{{\left( {k_{1}  - k_{2} } \right)\left( {k_{1}  - k_{3} } \right)}}{{k_{2} k_{3} }} \cdot e^{{ - k_{1}  \cdot t}} }  \\    {B_{t} } &  =  & {C_{1}  \cdot \frac{{k_{1} \left( {k_{1}  - k_{3} } \right)}}{{k_{2} k_{3} }} \cdot e^{{ - k_{1}  \cdot t}}  + C_{2}  \cdot \frac{{k_{2}  - k_{3} }}{{k_{3} }} \cdot e^{{ - k_{2}  \cdot t}} }  \\    \begin{gathered}   C_{t}  \hfill \\   D_{t}  \hfill \\  \end{gathered}  & \begin{gathered}    =  \hfill \\    =  \hfill \\  \end{gathered}  & \begin{gathered}    - C_{1}  \cdot \frac{{k_{1} }}{{k_{3} }} \cdot e^{{ - k_{1}  \cdot t}}  - C_{2}  \cdot \frac{{k_{2} }}{{k_{3} }} \cdot e^{{ - k_{2}  \cdot t}}  - C_{3}  \cdot e^{{ - k_{3}  \cdot t}}  \hfill \\   C_{1}  \cdot e^{{ - k_{1}  \cdot t}}  + C_{2}  \cdot e^{{ - k_{2}  \cdot t}}  + C_{3}  \cdot e^{{ - k_{3}  \cdot t}}  + C_{4}  \hfill \\  \end{gathered}   \\   \end{array}  $$

Using as initial conditions A_t=0_ = A_0_, B_t=0_ = 0, C_t=0_ = 0, and D_t=0_ = 0 leads to a linear system of four equations in the four scalar unknowns C_1_, C_2_, C_3_, and C_4_ the resolution of which leads to:

T $$ C_{1}  = A_{0}  \cdot \frac{{k_{2}  \cdot k_{3} }}{{\left( {k_{2}  - k_{1} } \right) \cdot \left( {k_{1}  - k_{3} } \right)}} $$

U $$ C_{2}  = A_{0}  \cdot \frac{{k_{1}  \cdot k_{3} }}{{\left( {k_{1}  - k_{2} } \right) \cdot \left( {k_{2}  - k_{3} } \right)}} $$

V $$ C_{3}  = A_{0}  \cdot \frac{{k_{1}  \cdot k_{2}  \cdot \left( {k_{2}  - k_{1} } \right)}}{{\left( {k_{1}  - k_{2} } \right) \cdot \left( {k_{1}  - k_{3} } \right) \cdot \left( {k_{2}  - k_{3} } \right)}} $$

W $$ C_{4}  = A_{0}  \cdot \frac{{k_{2}  \cdot k_{3}  \cdot \left( {k_{2}  - k_{3} } \right) - k_{1}  \cdot k_{3}  \cdot \left( {k_{1}  - k_{3} } \right) + k_{1}  \cdot k_{2}  \cdot \left( {k_{1}  - k_{2} } \right)}}{{\left( {k_{1}  - k_{2} } \right) \cdot \left( {k_{1}  - k_{3} } \right) \cdot \left( {k_{2}  - k_{3} } \right)}} $$

Finally, introducing Eqs. T, U, V and W into Eq. syst. C and re-arranging leads to Eqs. 8, 9, 10 and 11 above.
